# Elevated interleukin-12B is associated with increased seizure susceptibility: insights from two-sample Mendelian randomization and *in vivo* experiment

**DOI:** 10.3389/fneur.2025.1706857

**Published:** 2026-01-13

**Authors:** Yunyun Lu, Yan Li, Aijun Feng, Yanyan Zhang, Faqiang Li, Feng Chen, Shuaishuai Wang, Yu Wang

**Affiliations:** 1 Department of Neurology, The First Affiliated Hospital of Anhui Medical University, Hefei, China; 2 Department of Neurology, Xuzhou Cancer Hospital, Xuzhou, China; 3 Department of Neurology, Wuhu Second People’s Hospital, Wuhu, China; 4 Department of Neurology, Anhui Public Health Clinical Center, Hefei, China

**Keywords:** anti-inflammatory therapy, epilepsy, interleukin-12B, Mendelian randomization, seizure susceptibility

## Abstract

**Objective:**

To evaluate the causality between interleukins (ILs) and epilepsy using Mendelian randomization (MR) analysis. Furthermore, *in vivo* experiments were conducted to validate the results.

**Methods:**

All summary datasets for MR analysis were sourced from publicly available genome-wide association studies. MR analysis was utilized to assess the causal relationship between ILs and generalized epilepsy (GE). Comprehensive sensitivity analyses were carried out to verify the robustness of the findings. Additionally, seizure threshold, seizure score, and video electroencephalography recordings were conducted to evaluate the effect of IL-12B on seizure susceptibility.

**Results:**

The MR results revealed a causal effect of IL-12B on GE (IVW: *β* = 0.17, OR = 1.18, 95% CI = 1.05–1.34, *p* = 0.007). Estimated effects derived from supplementary methods were consistent with this finding. The robustness of these results was confirmed by sensitivity tests. Mice pretreated with IL-12B exhibited a significantly decreased seizure threshold, higher seizure scores, shortened latency to SE, and reduced survival probability compared with those pretreated with saline.

**Conclusion:**

Our study demonstrated that elevated serum levels of IL-12B are associated with an increased risk of GE. The results were validated through *in vivo* experiments. These results underscore the crucial role of targeted anti-inflammatory therapy in the treatment of epilepsy.

## Introduction

Epilepsy is a common chronic brain disorder characterized by a long-lasting predisposition to recurrent epileptic seizures (1). In 2021, approximately 52 million people with active epilepsy, with over 80% of the burden concentrated in low-income to middle-income countries (2). This underscores the need for better treatment and prevention of epilepsy. Though more than 20 antiseizure medications (ASMs) are currently available, around one-third of patients with epilepsy are refractory to drug treatment (3). This is because ASMs aim to suppress seizures but do not target the underlying epileptogenesis (1, 3, 4). Thus, gaining a comprehensive understanding of the complex pathogenesis of epilepsy is crucial for its effective treatment and prognosis.

Numerous studies have suggested that inflammatory pathways are involved in the epileptogenesis (5–8). Abnormal activation of critical inflammatory processes contributes to transforming a normal brain into an epileptic brain (7, 8). Elevated levels of inflammatory factors result in alterations in neurotransmitters and receptors, oxidative stress, and ion concentrations (9), leading to an imbalance between excitation and inhibition within the neuronal network (10). Interleukins (ILs) play a crucial role in the neuro-inflammatory pathogenesis of neurological diseases (11). Abnormal plasma levels of inflammatory biomarkers, such as IL-1, IL-2, IL-6, and IL-10, have been observed in patients with epilepsy independent of the underlying etiology (12). In epilepsy patients, serum levels of IL-1β, IL-7, IL-12, and IL-17 were markedly higher than those in healthy controls, and in refractory epileptic patients, these cytokines’ levels showed a positive correlation with National Hospital Seizure Severity Scale scores (13). IL-1β, IL-6, and TNF-*α* generate downstream inflammatory responses that damage the blood–brain barrier (BBB), increase the infiltration of serum proteins and immune cells into the brain, and promote neuronal hyperexcitability and seizure susceptibility (9, 14). In animal models of epilepsy, anti-inflammatory treatments have been shown to ameliorate neuronal loss and seizure susceptibility during epileptogenesis (7, 8). Thus, further clarification of the relationship between ILs and epilepsy is needed, which may potentially facilitate the identification of effective pharmaceutical targets for treatment.

However, previous observational studies may be susceptible to confounding factors (15), resulting in an ambiguous causal relationship between epilepsy and ILs. Mendelian randomization (MR) analysis can detect the causal association between exposure and outcome by utilizing single nucleotide polymorphisms (SNPs) as instrumental variables (IVs) (16, 17). This approach effectively reduces confounding factors and avoids bias arising from reverse causation (18, 19). Genome-wide association studies (GWAS) have identified 1,000 of genetic variants associated with a variety of complex diseases, thereby promoting the widespread application of MR analysis (20). Currently, MR studies have been employed to investigate the causal effects of multiple risk factors on epilepsy (21, 22).

In this study, we utilized MR analysis to evaluate the causality between ILs and epilepsy. Subsequently, *in vivo* experiments were conducted to validate the findings. These efforts enhance our comprehensive understanding of the association between ILs and epilepsy and provide potential therapeutic strategies.

## Materials and methods

### Study design

**Figure 1:**
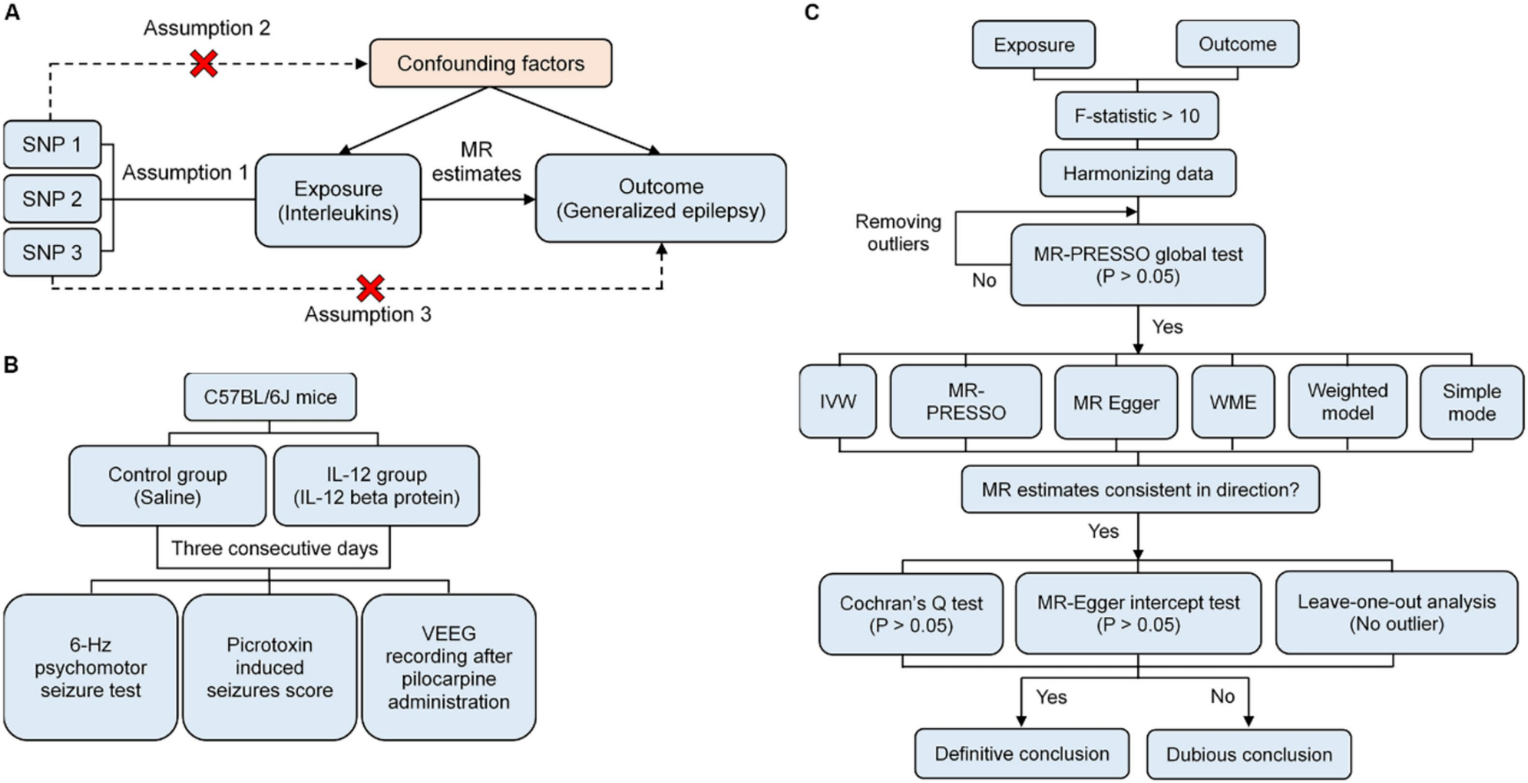
Schematic overview of the study. **(A)** Three fundamental assumptions underlying the MR study. **(B)** Flow diagram illustrating the *in vivo* experiment. **(C)** Flow diagram depicting the MR analysis process.

This study comprises two components. First, a two-sample MR analysis was performed to explore the causal relationship between ILs and epilepsy (Figure 1A). The MR study adheres to three basic assumptions (19), ensuring the validity of IVs: (1) the selected IVs are associated with ILs; (2) the IVs are not affected by confounding factors; (3) the IVs influence the epilepsy only through ILs, without influencing epilepsy via other pathways. This study follows the STROBE-MR guidelines (23). Second, *in vivo* experiments were carried out, and C57BL/6J mice were selected to validate the results obtained from the MR analysis (Figure 1B).

### Data sources

Two publicly available GWAS datasets were utilized for MR analysis. The datasets for ILs were obtained from a genome-wide protein quantitative trait locus study of 91 circulating inflammatory proteins in 14,824 participants from 11 cohorts (24). The datasets for generalized epilepsy (GE) were obtained from the FinnGen Consortium R12 release, encompassing 1,690 epilepsy patients and 484,697 control subjects (25). The diagnosis of epilepsy in FinnGen is based on G40 in the 10th edition of the International Classification of Diseases. Detailed information regarding the datasets can be found in the original articles and Supplementary Table 1. Ethics approval was not required for the current study, as these datasets have been ethically approved and informed consent has been obtained from participants in the original research.

### Instrumental variables selection

To acquire high-quality IVs for each exposure, a series of quality control measures were applied. First, we screened SNPs using a significance threshold of *p*-value < 5e−8. If no SNPs were extracted from ILs, a looser threshold of *p*-value < 5e−6 was applied (26, 27). Second, clumping thresholds (kb = 10,000; *r*^2^ = 0.001) were set to screen for independent SNPs that were not affected by linkage disequilibrium (28). Third, we assessed the strength of IVs by calculating *F*-statistics, and SNPs with *F*-value < 10 were excluded to avoid weak IVs (18, 28). Next, palindromic SNPs were excluded to prevent alleles from affecting the causal outcomes (29). Finally, outliers were detected using the MR-PRESSO global test, and SNPs were removed until the *p*-value for the global test was non-significant (*p*-value > 0.05) (30, 31). Consequently, the remaining SNPs were used for MR analysis.

### Mendelian randomization analysis

The MR analysis was conducted utilizing the inverse variance weighted (IVW) as the primary method to explore the causality between ILs and GE, the Wald ratio was used to evaluate causal effects when only one SNP was available (28, 32). MR-PRESSO, MR-Egger, simple mode, weighted mode, and weighted median were included as complementary methods. MR-PRESSO can detect pleiotropic outliers, particularly when horizontal pleiotropy is present in fewer than 50% of the IVs (31). MR-Egger can detect and correct for genetic pleiotropy. The influence of pleiotropy on the estimation of causal effect can be ignored when the *p*-value of pleiotropy test is greater than 0.05 (33). WME can yield robust results when more than 50% of the weights are derived from invalid IVs, while the weighted and simple mode require valid IVs to estimate the true causal effect (34, 35). If all SNPs are valid instrumental variables, the IVW method is more effective than other methods. Therefore, the IVW was used as the primary method to analyze the causality between ILs and GE. The results were considered consistent if all methods indicated the same direction of the effect (Figure 1C).

### Sensitivity analysis

We performed a series of sensitivity analyses to ensure the robustness of results. MR-Egger regression intercepts were carried out to evaluate the horizontal pleiotropy, and Cochrane *Q*-tests were performed to assess the heterogeneity (36, 37). A *p*-value > 0.05 indicated the absence of pleiotropy or heterogeneity among SNPs. The causal direction between ILs and GE was determined using Steiger test, which evaluated whether direction was consistent with the causal effect (38). The leave-one-out method was used to evaluate the impact of each SNP on the results (39).

### *In vivo* experiments

#### Animal

Adult male C57BL/6 J mice aged 8–10 weeks and weighing 22–25 g were provided by the Experimental Animal Center of Anhui Medical University. Mice were housed in a specific pathogen-free environment, maintained at a temperature of 21–25 °C, and subjected to a 12-h light–dark cycle. The animals had free access to food and water. This study complied with national legislation regarding the Care and Use of Animals. All experimental protocols were approved by the Ethics Committee of Anhui Medical University (No. 20252055).

#### Experimental design

Mice were randomly assigned into two groups. (1) Control group: mice received an intraperitoneal (i.p.) injected of saline (10 mL/kg); (2) IL-12B group: mice were administered an injection of IL-12 beta protein (mouse, HEK293, His; 1 μg/mouse/day, i.p., MedChemExpress, USA) for three consecutive days. No significant severe adverse effects or toxicity were observed following three injections of 1 μg of IL-12 alone (40). IL-12 has long been considered an effective agent in enhancing the anti-tumor immunological response for glioma therapy (41, 42). Seizure susceptibility was evaluated 6 h after the last IL-12 administration using the 6-Hz psychomotor seizure test and picrotoxin-induced seizure scores.

#### 6-Hz psychomotor seizure test

The 6-Hz psychomotor seizure test was conducted as previously described (7). Before stimulation, 50 μL of 0.5% tetracaine hydrochloride ophthalmic solution was applied to bilateral corneas. The electrodes were coated with a thin layer of electrode gel. A current (0.2 ms pulse-width, 3 s duration, and 6 pulses/s frequency) was delivered using a constant-current device (Ugo Basile ECT Unit, Italy). The CC50 (the intensity of current required to induce 6-Hz seizures in 50% of the animals) was recorded as a parameter for seizure susceptibility. In a preliminary experiment, 20 mA was identified as the CC50 for wild-type C57BL/6 mice. A 20 mA current was applied to the first mouse in each group, and then the current was adjusted by increasing or decreasing it at 0.06-log intervals. After stimulation, the mouse was placed in a transparent cage for behavioral observation. The behaviors of psychomotor seizures were characterized by stun, rearing, forelimb clonus, twitching of vibrissae, and an elevated tail. The mouse was scored as successful seizures if it exhibited behavioral seizures for at least 10 s.

#### Picrotoxin induced seizures

Mice were injected with picrotoxin (2.0 mg/kg, i.p., Topscience, China). Subsequently, seizure scores were recorded at 5-min intervals over a 30-min period. Behavioral seizures were scored as previously described (7). In brief, stage 1: immobility; stage 2: forelimb stretch; stage 3: recurrent circling or scratching; stage 4: repeated rearing; stage 5: repeated behavior of stages 2–4; stage 6: generalized rigidity; and stage 7: death.

#### Video electroencephalography (VEEG) recording

Mice were anesthetized with isoflurane (3.5% induction, 1.5% maintenance) and fixed on a stereotaxic apparatus (RWD Life Science, China). After disinfection with 1% iodophor, a rostrocaudal incision was made on the skull skin. 0.3% medical hydrogen peroxide was applied to remove the soft tissue and expose the bregma and posterior fontanelle. Small holes were drilled on the skull over the hippocampus according to the Mouse Brain in Stereotaxic Coordinates (2nd edition, Paxinos and Franklin) (43): 2.00 mm posterior to bregma, ±2.00 mm lateral, and 0.20 mm ventral to the skull. Two epidural recording electrodes (Nanjing Greathink Medical Technology, Nanjing, China) were bilaterally implanted. Another two electrodes, positioned over the cerebellum on the skull, were used as ground and reference electrodes. Two copper wires were inserted into the cervical muscles to record electromyography. These electrodes were fixed with screws and dental acrylic cement. Animals were injected with ampicillin (100 mg/kg, i.p., Kelun Pharmaceutical Company, China) to prevent infection for 3 days.

After a 7-day recovery period, the mice were subjected to VEEG recording using a multi-channel EEG recording system (Nanjing Greathink Medical Technology, China). Before recording, mice were allowed to habituate to the test cage for 24 h. Six hours after the last IL-12 injection, the EEG baseline was recorded for 60 min. Then the mice were pretreated with scopolamine methyl nitrate (1 mg/kg, i.p., Glpbio, USA) to prevent peripheral cholinergic effects. After 30 min, pilocarpine hydrochloride (280 mg/kg, i.p., Topscience, USA) was injected to induce status epilepticus (SE). The VEEG was recorded for another 60 min. Behavioral seizures were scored as previously described (7): stage 1: chewing, face and vibrissae twitching; stage 2: head nodding, unilateral limb clonus; stage 3: bilateral limb clonus or mild whole-body convulsion; stage 4: whole-body convulsion with tail hypertension; stage 5: rearing and falling with whole-body rigidity, and stage 6: death. SE was defined as a seizure that reached stage ≥ 3 and repeated or prolonged behavioral seizures. Diazepam (2.5 mg/kg, i.p., Kelun Pharmaceutical Company, China) was administered 120 min after SE induction.

EEG signals were down-sampled at 500 Hz and band-pass filtered from 5 to 80 Hz with a 50-Hz notch filter. The latency to SE and survival probability were recorded to evaluate the seizure susceptibility between two groups.

### Statistical analysis

In the MR study, causal estimates were expressed as odds ratios (ORs) with 95% confidence intervals (95% CI). Two-sample MR analysis was conducted using R version 4.3.2 within R Studio (R Foundation for Statistical Computing, Vienna, Austria), employing the TwoSampleMR package (version 0.5.7) and the MR-PRESSO package (version 1.0.0) (31, 44). A conservative Bonferroni-corrected *p*-value threshold of 0.05/15 (3.33e−3) was established to assess the causal effect between ILs and GE (45). A *p*-value < 0.05 was considered suggestive significance.

In *in vivo* experiments, the distribution of all data was evaluated. Data are presented as mean ± SD or median (interquartile range [IQR]) for variables with normal or skewed distributions, respectively. Student’s *t*-test or Mann–Whitney *U*-test were used to analyze differences between groups. All graphs were generated using GraphPad Prism (version 8.4.2, USA). Images were processed using Adobe Photoshop CS6 (Adobe, USA). Statistics were analyzed using SPSS software (version 22.0; IBM Inc., USA).

## Results

### Instrumental variables selection

A total of 16 ILs were selected for MR analysis, including IL-1A, IL-2, IL-4, IL-5, IL-6, IL-7, IL-8, IL-10, IL-12B, IL-13, IL-17A, IL-17C, IL-18, IL-20, IL-24, and IL-33. Among these, SNPs derived from IL-2, IL-4, IL-5, IL-17, IL-20, IL-24, and IL-33 were identified using a significance threshold of *p*-value < 5e−6. Subsequently, 205 SNPs associated with all these ILs were identified. The *F*-statistics of the included IVs were all greater than 10 (Min = 20.84, Max = 1423.92), indicating that the absence of weak IVs. However, IL-7 was excluded for the final MR analysis, because no SNP was detected in the IL-7 dataset after merging with the GE dataset (Supplementary Table 2).

### Causality of interleukins on generalized epilepsy

**Figure 2:**
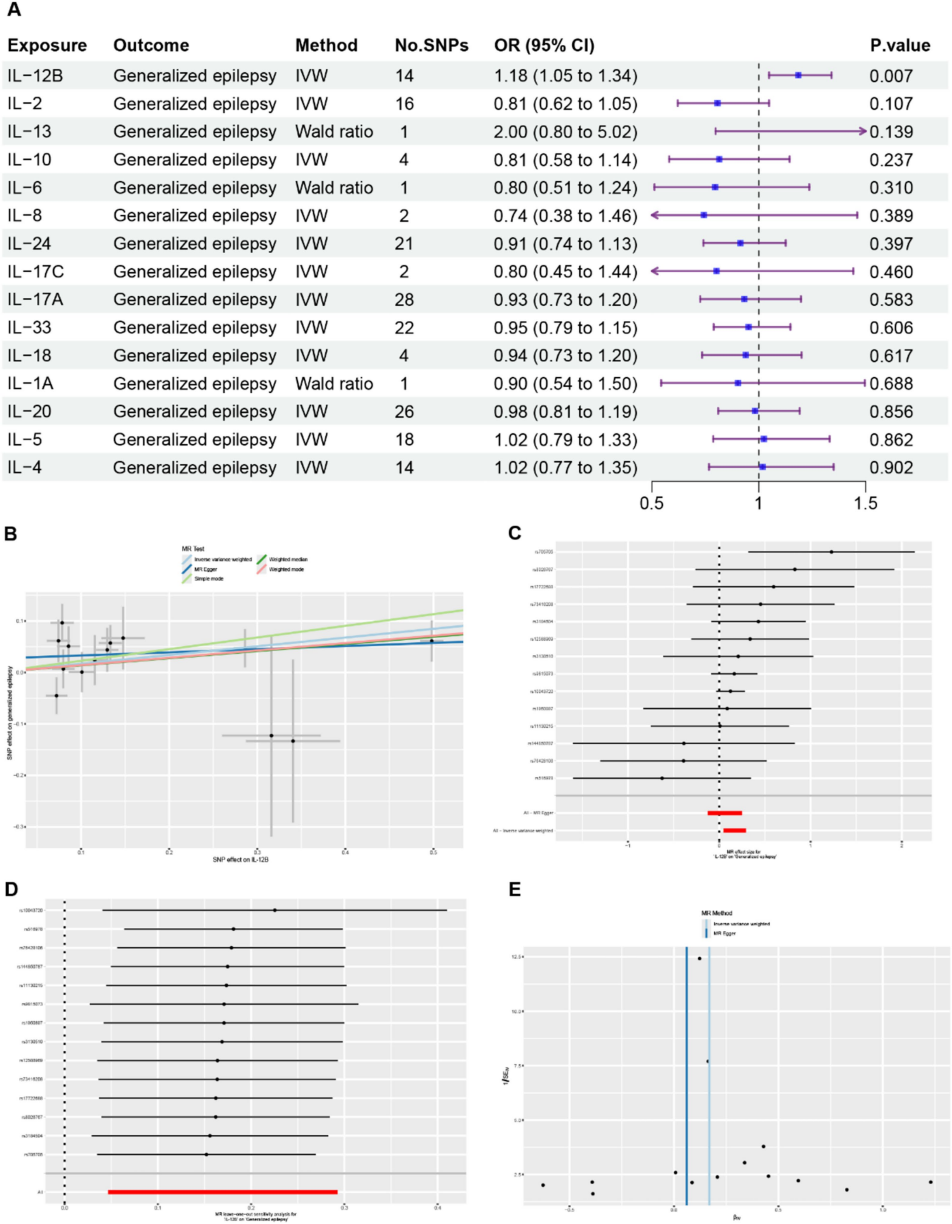
Comprehensive results of the MR analysis. **(A)** Causal estimates presented as odds ratios (ORs) and 95% confidence intervals for the effect of ILs on GE. **(B)** Scatter plots depicting the causal relationship between IL-12B and GE. **(C)** Detailed forest plots displaying the estimated MR effect size of IL-12B on GE. **(D)** Leave-one-out analysis assessing the effect of IL-12B on GE. **(E)** Funnel plot evaluating the potential heterogeneity between IL-12B and GE.

In the MR analysis, 15 ILs were designated as the exposures, and GE was identified as the outcome. The results obtained from the MR analysis are presented in Figure 2A and Supplementary Table 3. Only IL-12B was found to be associated with GE. The results generated using IVW method suggested that elevated levels of IL-12B were associated with an increased likelihood of GE (IVW: *β* = 0.17, OR = 1.18, 95% CI = 1.05–1.34, *p* = 0.007) (Figures 2A–C). Estimated effects obtained from MR-PRESSO, MR-Egger, WME, weighted model, and simple mode were concordant with this finding (Supplementary Tables 3, 4).

### Sensitivity analysis

No outliers were detected by the MR-PRESSO global test and leave-one-out analysis, suggesting that the causal association between IL-12B and GE was not driven by a single SNP (Figure 2D, Supplementary Table 5). No evidence of heterogeneity was detected using the Cochran’s *Q* test and funnel plot (Figure 2E, Supplementary Table 6). Potential horizontal pleiotropy among the IVs were also not detected by MR-Egger regression intercept analysis and the MR-PRESSO global test (Supplementary Table 5). The results of the Steiger test demonstrated that IL-12B is more likely to be the causal factor for GE (Supplementary Table 7). In general, these results provide robust evidence that elevated levels of IL-12B are associated with an increased risk of GE.

### IL-12 beta protein administration increases seizure susceptibility

Mice received IL-12 beta protein administration (1 μg, i.p.) once daily for three consecutive days. Six hours after last injection, the 6-Hz seizure threshold, picrotoxin induced seizure scores, and VEEG recordings during SE were measured in the mice.

**Figure 3:**
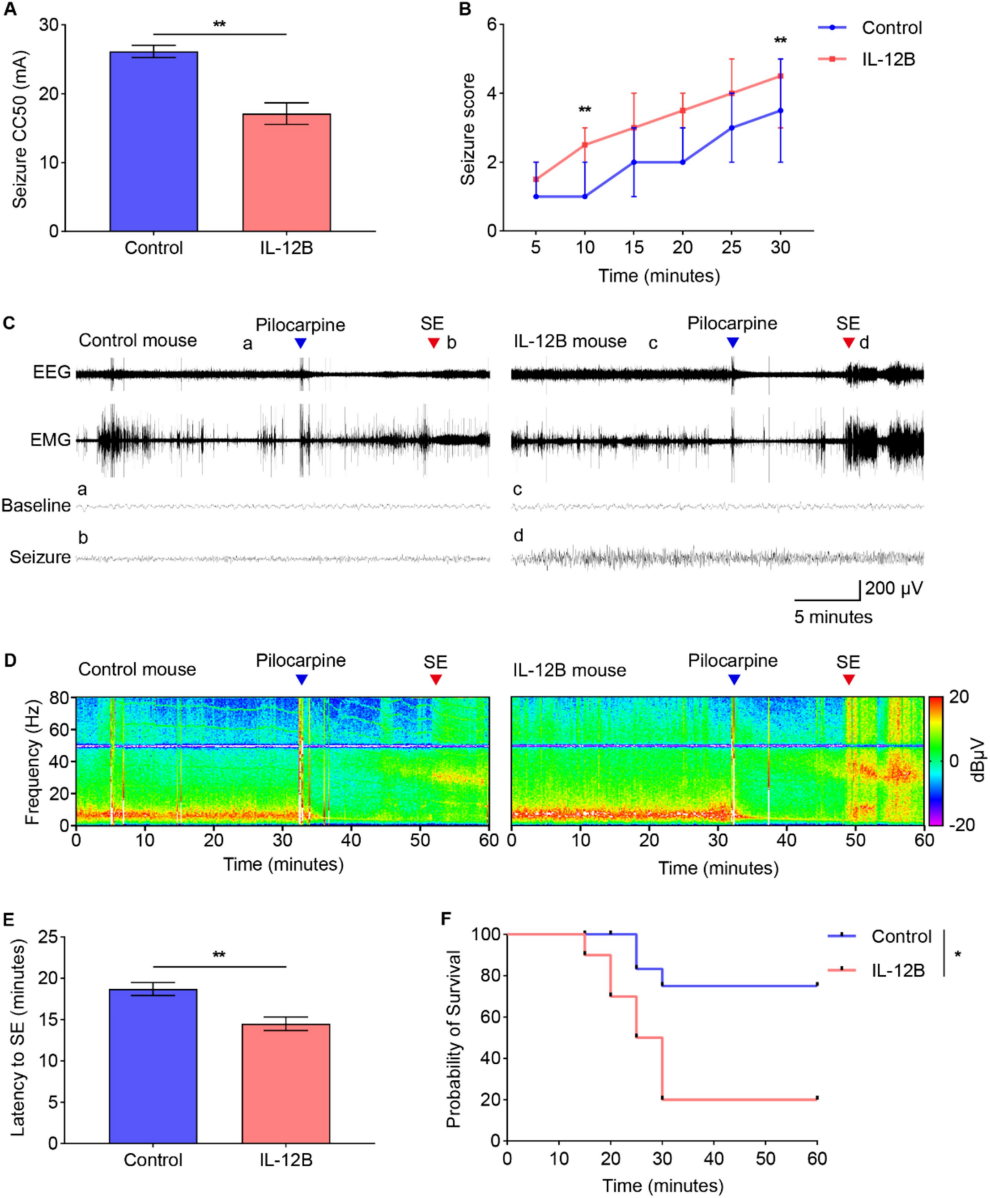
Administration of IL-12 beta protein increases seizure susceptibility. **(A)** Seizure susceptibility measured by 6-Hz seizure threshold test (*n* = 12 mice/group). **(B)** Seizure score within 30 min after picrotoxin administration (*n* = 6 mice/group). **(C)** A representative diagram illustrating EEG, EMG, baseline, and seizure patterns after pilocarpine administration. **(D)** A time-frequency spectrogram of EEG corresponding to the EEG in (C) (*n* = 10 mice/group). **(E)** Latency to SE after pilocarpine administration. **(F)** Survival probability within 60 min post pilocarpine administration (*n* = 10 mice/group). *p < 0.05, **p < 0.01 by Student’s *t* test (**A** and **E**), generalized linear models with Bonferroni test **(B)**, Kaplan–Meier analysis with Log Rank (Mantel-Cox) test **(F)**. Values are means ± SD (**A** and **E**), medians (IQR) **(B)**, rate **(F)**.

In the 6-Hz seizure threshold test, compared with the control group (26.15 ± 1.53 mA), a significantly decreased seizure threshold was observed in the IL-12B group (17.12 ± 2.71 mA) (Figure 3A). During the 30-min period after picrotoxin administration, the seizure scores were significantly higher in the mice treated with IL-12 beta protein than in those treated with saline (Figure 3B). VEEG signals were analyzed after pilocarpine administration (Figures 3C,D). The results demonstrated that the latency to SE was significantly shortened in the IL-12B group (14.50 ± 2.59 min) compared with the control group (18.70 ± 2.50 min) (Figure 3E). Additionally, the survival probability after pilocarpine-induced SE was drastically reduced in mice injected with IL-12 beta protein compared with the control group (Figure 3F). These findings collectively suggest that the mice exhibited more severe seizures following the administration of IL-12 beta protein.

## Discussion

In this study, MR analysis was conducted to investigate the causal relationship between ILs and GE, revealing that the elevated IL-12B is associated with an increased risk of GE. In the animal experiment, we observed that mice treated with IL-12 beta protein exhibited significantly increased seizure susceptibility compared with the control mice.

Various brain insults, such as infections, stroke, brain tumors, and traumatic brain injuries, contribute to the inflammatory etiopathogenesis of epileptic seizures (46–49). Abnormal activation of inflammatory processes transforms a normal brain into an epileptic brain (7, 46, 49). Additionally, brain inflammation induced by seizures is responsible for the recruitment of peripheral inflammatory cells in epileptic brains (50, 51). An increased biosynthesis of inflammatory mediators and upregulation of related receptors are observed in the epileptic brain (7, 8, 52, 53). In animal study, pilocarpine administration significantly elevated the levels of IL1β and IL-6 in the hippocampus compared with the control group (54). The overexpression of pro-inflammatory cytokines genes, such as IL-1β and TNF-*α*, was also observed in olfactory bulb tissue from patients with frontal lobe epilepsy (55). TNF-α has been demonstrated to be upregulated and overexpressed in brain regions involved in epileptic activity (56, 57). These results indicate that inflammatory processes within the brain play a crucial role in epileptogenesis.

It has been reported in animal study that systemic inflammation can reduce the threshold for seizures induced by pro-convulsive agents (58). In clinical study, a significant elevation of CCL2/MCP-1 in cerebrospinal fluid and plasma was observed in drug-resistant epilepsy patients (59). Serum IL-1β levels were correlated with the number of antiepileptic drugs used in children with epilepsy (60). Additionally, serum levels of HMGB-1, TLR4, and TNF-α were significantly higher in the severe epilepsy group than those in the control group and the mild epilepsy group (61). These results demonstrate that the serum cytokine levels are not only associated with epilepsy but also correlated with the disease severity. Thus, targeting inflammatory factors may potentially be beneficial to arresting epileptogenesis.

In our study, we found that the elevated levels of IL-12B are strongly associated with an increased risk of GE. IL-12 is a heterodimeric cytokine that belongs to the IL-12 cytokine family, which is involved in the transition from innate to adaptive immunity and implicated in the etiology of several autoimmune diseases (62). In the case of epilepsy, previous research has investigated microglial activation in epileptic models, revealing that IL-12 mRNA levels transiently increased following seizures induced by kainic acid (63). Compared with non-epileptic individuals, increased levels of IL-12 were observed in the epileptic brain, such as in the cerebral cortex and hippocampal region, which are responsible for epileptogenesis (64, 65). In epilepsy patients, the serum levels of IL-12 were significantly higher than those in healthy individuals. Moreover, in refractory epileptic patients, the levels of IL-12 exhibited a positive association with the scores on the National Hospital Seizure Severity Scale (13). IL-12 can induce microglia and macrophages to upregulate the expression of inducible nitric oxide synthase, thereby participating in the pathogenesis of neuroinflammatory diseases (66, 67). In addition, elevated IL-12 p70 levels within the brain may be correlated with the transient leukocyte changes observed following seizures, characterized by increased lymphocytes and neutrophils and a concurrent reduction in T cells (68). IL-12 p70 drives the proliferation and differentiation of T-cells, activates natural killer cells, and thus facilitates the induction of IFN-*γ* and TNF-*α*, through which it may inhibit the formation of new blood vessels (40, 64). Finally, due to the damage of the blood–brain barrier by inflammatory responses, increased extravasation of peripheral pro-inflammatory cells may contribute to local inflammation in epileptogenic lesions.

In our study, using *in vivo* experiments, we observed that mice treated with IL-12 beta protein exhibited significantly increased seizure susceptibility compared with the control mice. Specifically, IL-12 beta protein-treated mice showed a lower seizure threshold in the 6-Hz psychomotor seizure test, higher seizure scores induced by picrotoxin, a shorter latency to SE, and a lower survival probability after pilocarpine administration. The effects of systemic inflammation on seizures may be explained by the following mechanisms: (1) a peripheral inflammatory stimulus triggers a local brain inflammatory reaction similar to the response elicited in the periphery; (2) the epileptic brain displays an amplified and exaggerated response to a systemic inflammatory challenge (69). Finally, IL-12 beta facilitates neuroinflammation, modulates neurotransmitter systems, compromises neuroplasticity, and activates microglia through interactions with diverse signaling pathways, thereby exerting its proconvulsive effects (70).

## Limitations

Several limitations should be taken into account in our study. First, the MR analysis dose not fully replicate the conditions of real-world clinical trials, thus, the estimates of the relationship between ILs and GE may not precisely correspond to the results obtained in clinical settings (71). Secondly, the results obtained from plasma circulating ILs pose challenges in identifying the most effective therapeutic targets. Because different tissues exhibit distinct genetic regulatory mechanisms, focusing solely on blood biomarkers may not provide the comprehensive effects of ILs on GE. Finally, the MR results are limited by its predominant focus on European individuals. Therefore, additional research and validation are necessary to extrapolate these results to individuals of other ethnicities.

## Conclusion

In conclusion, our study revealed that elevated serum levels of IL-12B are associated with an increased risk of GE. Given that the results were validated through *in vivo* experiments, we demonstrated that mice treated with IL-12 beta protein exhibited significantly increased seizure susceptibility compared with the control mice. This research further elucidates the relationship between ILs and GE, highlighting the crucial role of targeted anti-inflammatory therapy in the treatment of epilepsy.

## Data Availability

The original contributions presented in the study are included in the article/Supplementary material, further inquiries can be directed to the corresponding author/s.
